# *Pseudomonas gilva* sp. nov., a Plant-Associated Bacterial Species with Growth-Promoting Traits and Partial Suppression of Peanut Southern Blight

**DOI:** 10.3390/microorganisms14102291

**Published:** 2026-10-08

**Authors:** Tengfei Ma, Haijiao Liu, Weizhen Zhao, Chenyang Du, Yafei Chen, Juan Liu, Yimeng Zheng, Yibo Wang, Risheng Xu, Xinyou Zhang

**Affiliations:** 1 Peanut Research Institute, Henan Academy of Agricultural Sciences, Zhengzhou 450002, China; 2 Henan Provincial Key Laboratory for Genetic Improvement of Oil Crops, Zhengzhou 450002, China; 3 Key Laboratory of Oil Crops in Huang-Huai-Hai Plains, Ministry of Agriculture, Zhengzhou 450002, China; 4 The Shennong Laboratory, Zhengzhou 450002, China

**Keywords:** *Pseudomonas*, polyphasic taxonomy, peanut southern blight, plant growth promotion, biocontrol

## Abstract

A rhizosphere-derived bacterial isolate, GYB4201^T^, was subjected to taxonomic and functional characterization. The strain consisted of Gram-stain-negative, strictly aerobic rods and was positive for both catalase and oxidase activities. Core-genome phylogeny placed GYB4201^T^ within the *Pseudomonas putida* group, in which it occupied an independent and well-supported branch. Pairwise ANI and dDDH values relative to the most closely related type strains were below the accepted species boundaries. Its phenotypic and chemotaxonomic profiles provided additional evidence for taxonomic distinction. The cellular fatty acid composition was dominated by C_16:0_, Summed Feature 8 and Summed Feature 3. Phosphatidylethanolamine, phosphatidylglycerol and diphosphatidylglycerol were the principal polar lipids, while ubiquinone-9 was the predominant respiratory quinone. Functional assays demonstrated phosphate and potassium solubilization, indole-3-acetic acid production, CAS-positive siderophore-related activity, and inhibitory activity against *Sclerotium rolfsii*. Pot experiments further showed that inoculation with GYB4201^T^ reduced peanut southern blight severity and promoted peanut growth. Positive but non-significant trends were observed in several root architectural traits. These taxonomic and functional data support the recognition of GYB4201^T^ as a novel species of the genus *Pseudomonas*, for which the name *Pseudomonas gilva* sp. nov. is proposed. The type strain is GYB4201^T^ (=CFCC 17085^T^ = ICMP 25013^T^).

## 1. Introduction

Peanut is an economically important oilseed and cash crop in China, with a cultivation area exceeding 4.87 million hectares [1]. Southern blight, caused by Sclerotium rolfsii, is a destructive soil-borne disease that seriously threatens peanut production [2]. The pathogen can persist in soil and infect host plants aggressively, making disease management difficult [2]. Control therefore often relies heavily on chemical fungicides. However, repeated fungicide application may result in environmental contamination, pesticide residues, disruption of soil microbial communities, and the emergence of resistant pathogen populations [3,4]. These limitations have increased interest in sustainable microbial approaches for disease suppression and plant growth promotion.

Members of the genus Pseudomonas are among the best-studied plant-associated bacteria with recognized biocontrol and plant growth-promoting potential [5]. Beneficial Pseudomonas strains can suppress soil-borne pathogens through antagonism and resource competition [6,7]. They may also improve plant performance by mobilizing poorly available nutrients, producing siderophores, and synthesizing phytohormone-related metabolites [6,7]. Several recently described Pseudomonas species have shown plant growth-promoting or biocontrol potential based mainly on in vitro antagonistic assays, plant-beneficial phenotypes, or genome mining [8,9,10]. However, direct validation at the plant level remains limited. Integrating taxonomic characterization with plant-level functional evaluation is therefore important for determining whether promising isolates can translate these traits into measurable effects in planta.

During the screening of plant-associated bacteria, strain GYB4201^T^ was identified as a promising isolate because it exhibited antagonistic activity against *S. rolfsii* and several plant-associated functional traits. In the present study, we characterized these traits in vitro and evaluated the effects of GYB4201^T^ on peanut southern blight, plant growth, and root system architecture in pot experiments. The taxonomic position of the strain was also examined using a polyphasic approach. This study therefore aimed to clarify the taxonomic status of GYB4201^T^ and evaluate its potential as a plant-associated bacterial resource for peanut.

## 2. Materials and Methods

### 2.1. Strain and Culture Conditions

Strain GYB4201^T^ was isolated from rhizosphere soil collected in Sichuan, China, on 5 April 2021 (26°22′44.13″ N, 101°37′55.35″ E). For bacterial isolation, soil samples were suspended in sterile water, serially diluted, and spread onto TSA agar plates [11]. After incubation at 28 °C for 2 days, morphologically distinct colonies were selected and purified by repeated streaking on fresh plates of the same medium. The purified strain was routinely cultured on TSA at 28 °C and preserved at −80 °C with a supplement of 20% (*v*/*v*) glycerol.

### 2.2. Genomic Data

The genome of strain GYB4201^T^ was sequenced using the Illumina NovaSeq PE150 platform by Novogene Co., Ltd. (Beijing, China). Raw reads were filtered to remove adapter sequences and low-quality reads. Clean reads were independently assembled using SOAPdenovo (version 2.04) [12,13], SPAdes [14], and ABySS [15], and the resulting assemblies were integrated using CISA [16]. The draft genome sequence of strain GYB4201^T^ was deposited in the NCBI database under accession number JBWGIG000000000. Genome sequences of reference-type strains included in this study were downloaded from the NCBI database. Genome quality was evaluated using CheckM [17], and only genomes with completeness ≥95% and contamination ≤5% were retained. A total of 68 high-quality genomes, including that of strain GYB4201^T^ and the selected reference-type strains, were used for subsequent phylogenomic and genome-based relatedness analyses (Table S1).

### 2.3. Phylogenetic Tree Construction

The 16S rRNA gene sequences of reference-type strains were downloaded from the NCBI database. The sequences were aligned with CLUSTAL W in MEGA X [18], and phylogenetic trees were reconstructed using the maximum-likelihood, neighbour-joining and maximum-parsimony methods. Bootstrap values were calculated from 1000 resamplings.

Genome-scale phylogeny was analysed under a Linux environment using the Up-to-date Bacterial Core Gene (UBCG) pipeline based on 92 conserved bacterial genes [19]. Core genes were identified, aligned and concatenated according to the standard UBCG workflow before phylogenomic reconstruction.

A concatenated phylogenomic tree was additionally reconstructed based on single-copy orthologous proteins. Briefly, the whole-genome sequences of strain GYB4201^T^ and 67 reference genomes included in this study were used for comparative analysis. Protein-coding sequences were predicted from all genomes using Prodigal (version 2.6.3) [20]. Orthologous gene clustering was then carried out with OrthoFinder (version 2.5.5) [21] under default settings, and 765 single-copy orthologous proteins shared by the 68 genomes were identified. The amino acid sequences of these orthologues were concatenated into a single alignment, which was subsequently used for phylogenomic reconstruction with FastTree (version 2.1.11) [22]. The resulting tree was visualized and edited using iTOL (version 7) [23].

### 2.4. Genome-Based Metric Analyses

Genome-based relatedness was assessed using average nucleotide identity (ANI) and digital DNA–DNA hybridization (dDDH), which were used as genomic indices for species delineation [24]. Average nucleotide identity based on BLAST (ANIb) was calculated using pyani (version 0.2.9) [25], whereas digital DNA–DNA hybridization (dDDH) values were estimated using formula 2 of the Genome-to-Genome Distance Calculator (GGDC; http://ggdc.dsmz.de).

### 2.5. Chemotaxonomy and Physiology

Chemotaxonomic analyses of strain GYB4201^T^ were conducted using standard procedures. Cellular fatty acids were extracted from cells grown on TSA and analysed with the Sherlock Microbial Identification System (MIDI) [26]. Polar lipids were extracted from freeze-dried biomass and separated by two-dimensional thin-layer chromatography [27]. Respiratory quinones were extracted from lyophilized cells and identified by HPLC [27,28].

Physiological characterization included the determination of growth ranges for temperature, pH and NaCl concentration. Gram staining, oxidase activity, catalase activity and anaerobic growth were examined by routine procedures [11]. Carbon source utilization patterns were assessed using Biolog GN2 according to the manufacturer’s instructions.

### 2.6. In Vitro Functional Assays

Several in vitro traits potentially related to plant-associated interactions were examined for strain GYB4201^T^. Inorganic phosphate-solubilizing activity was assessed on insoluble phosphate agar plates, and the formation of a clear halo around the colony was taken as preliminary evidence of phosphate solubilization [29]. Potassium-solubilizing activity was evaluated on Aleksandrov agar, with the formation of a clear halo around the colony considered a positive result. Siderophore-related activity was qualitatively screened using chrome azurol S (CAS) agar, with the formation of an orange-yellow halo recorded as a positive CAS reaction [30].

Indole-3-acetic acid (IAA) production was determined using the Salkowski colorimetric assay [31]. Briefly, the strain was inoculated into ISP1 broth and incubated in the dark on a rotary shaker for 72 h. After centrifugation, 0.5 mL of the culture supernatant was mixed with an equal volume of Salkowski reagent, and the development of a pink colour was recorded as positive for IAA production.

Antagonistic activity against *Sclerotium rolfsii* was evaluated by dual-culture assay on TSA plates [32]. A 5 mm agar plug taken from the actively growing margin of the fungal colony was placed at the centre of the plate, and strain GYB4201^T^ was inoculated away from the fungal plug. TSA plates inoculated with the fungus alone served as controls. All assays were performed in triplicate and incubated at 25 °C.

### 2.7. Pot Experiment for Peanut Growth Promotion

A pot experiment was conducted to evaluate the growth-promoting effect of strain GYB4201^T^ on peanut. Peanut seeds of cultivar Yuhua 37 were surface-disinfected with 75% ethanol for 1 min and subsequently rinsed three times with sterile distilled water, following a previously established protocol for peanut pot experiments [33]. Seeds were grown in a substrate consisting of seedling-growing medium, vermiculite and perlite at a ratio of 3:2:1. Strain GYB4201^T^ was cultured in TSB at 28 °C for 24 h, harvested by centrifugation, and resuspended in sterile water. The bacterial suspension was adjusted to OD 600 = 0.25. Each plant was inoculated with 10 mL of bacterial suspension by root drenching, while control plants received an equal volume of sterile water. The experiment consisted of a non-inoculated control and a bacterial inoculation treatment, with five biological replicates per treatment. At 21 d after inoculation, plant height, shoot fresh weight, shoot dry weight, root fresh weight and root dry weight were measured [34]. Treatment effects were expressed as response ratios, with 95% confidence intervals calculated using R software (version 4.4.3).

Root morphology was analysed at the end of the growth-promotion experiment. Roots were carefully removed from the substrate, washed gently with water to remove adhering soil particles, and scanned using a root scanner coupled with WinRHIZO (version 4.0B, Regent Instruments Inc., Quebec City, QC, Canada). The scanned images were analysed to determine total root length, average root diameter, total root surface area and total root volume [35].

### 2.8. Pot Experiment for the Control of Peanut Southern Blight

A pot experiment was conducted to evaluate the suppressive effect of strain GYB4201^T^ against peanut southern blight caused by *Sclerotium rolfsii*. Strain GYB4201^T^ was prepared as described above, and the bacterial suspension was adjusted to OD 600 = 0.1 before inoculation.

For bacterial treatment, peanut seedlings at 14 days after sowing and showing uniform growth were selected, and each seedling was inoculated with 10 mL of the bacterial suspension using a root-drenching method. Control seedlings received an equal volume of sterile water. After 24 h, both the bacterial treatment and pathogen-control plants were inoculated with *S. rolfsii* by placing eight wheat grains colonized by the pathogen evenly around the stem base of each plant.

Plants were maintained under greenhouse conditions, and disease development was monitored for 10 d after pathogen inoculation. Disease severity was assessed using a 0–4 disease rating scale, while disease index and control efficacy were calculated from the disease rating data [32].

## 3. Results

### 3.1. Genome-Based Phylogenetic Analysis

In the taxonomy of the genus *Pseudomonas*, 16S rRNA gene sequences often provide insufficient phylogenetic resolution for closely related taxa (Figure S1). Therefore, the phylogenetic position of strain GYB4201^T^ was further examined using two genome-based approaches, namely a UBCG phylogenomic tree based on 92 core genes and a concatenated phylogenomic tree reconstructed from 765 single-copy orthologous proteins (Figure 1 and Figure S2). The two genome-based trees showed highly similar topologies and provided a clearer resolution of the phylogenetic relationships among closely related *Pseudomonas* species than the 16S rRNA gene tree.

Strain GYB4201^T^ showed the highest 16S rRNA gene sequence similarities to *Pseudomonas bananamidigenes* BW11P2^T^ (99.05%), *Pseudomonas moorei* RW10^T^ (98.90%), *Pseudomonas botevensis* COW3^T^ (98.70%), *Pseudomonas eucalypticola* NP-1^T^ (98.60%) and *Pseudomonas mohnii* DSM 18327^T^ (98.60%). However, the phylogenomic analyses did not fully agree with the 16S rRNA gene-based affiliations. In both the UBCG tree and the concatenated phylogenomic tree, strain GYB4201^T^ clustered with *P. eucalypticola* within the *P. putida* group, whereas *P. bananamidigenes*, *P. moorei*, *P. botevensis* and *P. mohnii* were placed in the *P. jessenii* group. These results indicate that high 16S rRNA gene sequence similarity does not necessarily reflect overall genome-level relatedness in the genus *Pseudomonas* and further highlight the limited discriminatory power of the 16S rRNA gene for resolving closely related taxa within this genus [36,37].

In both genome-based trees, strain GYB4201^T^ formed a stable and independent lineage within the *P. putida* group, supporting its assignment as a distinct species-level taxon in the genus *Pseudomonas*.

### 3.2. Genome Relatedness Based on ANI and dDDH

Average nucleotide identity (ANI) and digital DNA–DNA hybridization (dDDH) values were calculated between strain GYB4201^T^ and its closely related type strains to further assess its species-level taxonomic status (Table 1). All ANI and dDDH values were below the accepted thresholds for species delineation, indicating that strain GYB4201^T^ represents a distinct novel species of the genus *Pseudomonas*.

The highest ANI and dDDH values were obtained between GYB4201^T^ and *P. eucalypticola*, but these values were still well below the recommended cut-off values for species definition. Lower ANI and dDDH values were observed with *P. bananamidigenes*, *P. moorei*, *P. botevensis* and *P. mohnii*. This pattern is consistent with the genome-based phylogenetic analyses, which placed GYB4201^T^ together with *P. eucalypticola* in the *P. putida* group rather than with the taxa assigned to the *P. jessenii* group. Therefore, the genome-relatedness data provide additional support for the assignment of GYB4201^T^ as a novel species of the genus *Pseudomonas*.

### 3.3. Phenotypic, Physiological and Chemotaxonomic Characteristics

The major polar lipids of strain GYB4201ᵀ were phosphatidylethanolamine, phosphatidylglycerol and diphosphatidylglycerol (Figure S3), a profile consistent with those commonly reported for species of the genus *Pseudomonas* [38,39]. Several additional phospholipids and unidentified lipids were also detected, providing supplementary chemotaxonomic information for strain GYB4201^T^. The predominant respiratory quinone was Q-9, which was similar to those reported for related *Pseudomonas* species and supported its assignment to the genus *Pseudomonas*.

The major fatty acids of strain GYB4201^T^ were C_16:0_, Summed Feature 8 and Summed Feature 3 (Table S2). Although the overall fatty acid profile was similar to those of related species, GYB4201^T^ could be differentiated from *P. moorei*, *P. mohnii* and *P. eucalypticola* by differences in the relative amounts of C_10:0_ 3-OH, C_12:0_ 3-OH, C_16:0_, C_17:0_ cyclo and C_19:0_ cyclo *ω*8*c*, as well as by the occurrence of Summed Feature 3 and Summed Feature 8 instead of the corresponding unsaturated fatty acids detected as separate components in some related strains. Fatty acid data for *P. bananamidigenes* and *P. botevensis* were unavailable, and therefore these two strains were not included in the chemotaxonomic comparison.

In addition, strain GYB4201^T^ differed from its closest related type strains in several physiological and biochemical traits, including NaCl tolerance and substrate utilization patterns (Table 2). Taken together, these chemotaxonomic and phenotypic characteristics clearly distinguished strain GYB4201^T^ from its closest relatives and supported its recognition as a novel species of the genus *Pseudomonas*.

### 3.4. Plant-Associated Functional Traits

Strain GYB4201^T^ exhibited several in vitro traits associated with plant–microbe interactions. It showed positive activities for inorganic phosphate solubilization, potassium solubilization, indole-3-acetic acid production and siderophore production (Figure S4). In dual-culture assays, GYB4201^T^ also displayed antagonistic activity against *Sclerotium rolfsii*, as evidenced by the inhibition of mycelial growth on TSA plates (Figure S4). These results indicate that strain GYB4201^T^ possesses multiple plant-associated functional traits and a potential suppressive effect against the peanut southern blight pathogen.

### 3.5. Growth-Promoting Effects on Peanut

At 21 days after inoculation, GYB4201^T^-treated peanut seedlings displayed a visibly more vigorous phenotype than the non-inoculated controls (Figure 2A). Quantitative analysis showed higher values for all measured growth traits, with increases of 33.1% in plant height, 111.9% in shoot fresh weight, 70.0% in shoot dry weight, 50.7% in root fresh weight and 52.4% in root dry weight (Figure 2C). Root system images also indicated a generally more developed root architecture following inoculation (Figure 2B). Mean total root length, average root diameter, total root surface area and total root volume were 16.1%, 6.5%, 23.8% and 31.6% higher, respectively, in GYB4201^T^-treated plants than in the controls; however, these differences were not statistically significant. Thus, GYB4201^T^ markedly promoted several biomass-related growth traits, whereas its effects on root architectural parameters were expressed primarily as positive numerical trends.

### 3.6. Suppressive Effect Against Peanut Southern Blight

The suppressive effect of strain GYB4201^T^ against peanut southern blight was evaluated in a greenhouse pot experiment. Compared with the pathogen-inoculated control, plants treated with GYB4201ᵀ generally showed less severe symptom development (Figure 3A), although variation in disease severity was observed among individual plants within both treatments. Quantitative assessment showed that the disease index decreased from 77.5 in the control to 50.0 in the GYB4201^T^-treated group, corresponding to a control efficacy of 35.48% (Figure 3B). These results indicate that strain GYB4201^T^ exerted a partial suppressive effect against peanut southern blight under greenhouse conditions.

## 4. Discussion

Strain GYB4201^T^ represents a distinct species-level lineage within the genus *Pseudomonas*. Although its 16S rRNA gene showed high sequence similarity to several previously described *Pseudomonas* species, genome-scale phylogenetic analyses consistently placed GYB4201^T^ within the *P. putida* group and identified *P. eucalypticola* as its closest phylogenomic relative. This discrepancy between 16S rRNA gene-based and genome-based relationships further illustrates the limited discriminatory power of the 16S rRNA gene for species-level resolution within *Pseudomonas* [36,37]. The markedly lower ANI and dDDH values relative to established species thresholds [40,41], together with differential phenotypic and chemotaxonomic characteristics, provide robust support for the recognition of GYB4201^T^ as a novel species.

Beyond its taxonomic novelty, GYB4201^T^ displayed a combination of traits commonly associated with beneficial plant–microbe interactions, including phosphate and potassium solubilization, a positive reaction for indolic compounds, siderophore production and antagonistic activity against *S. rolfsii*. The antagonistic activity of GYB4201ᵀ against *S. rolfsii* may involve antibiosis, nutrient competition, or antifungal metabolites; however, the specific active compounds remain to be identified. Consistent with these in vitro phenotypes, inoculation with GYB4201^T^ enhanced several peanut growth traits under pot conditions, particularly shoot and root biomass. These findings indicate that GYB4201^T^ is not only taxonomically distinct but also possesses biologically relevant traits associated with plant performance.

Nevertheless, the functional effects observed in this study should be interpreted cautiously. Although several root architectural parameters showed numerical increases following bacterial inoculation, these differences were not statistically significant. In addition, the reduction in disease index corresponded to a control efficacy of 35.48%, indicating partial rather than complete suppression of disease. The current experiments were conducted under controlled pot conditions; therefore, further studies are needed to determine rhizosphere colonization dynamics, clarify the mechanisms underlying plant growth promotion and pathogen suppression, and evaluate the stability of these effects under field conditions.

## 5. Conclusions

Strain GYB4201^T^ represents a novel species of the genus *Pseudomonas*, as supported by phylogenomic analyses, genome relatedness indices, and distinctive phenotypic and chemotaxonomic characteristics. In addition to its taxonomic novelty, GYB4201^T^ exhibited several plant-associated functional traits, promoted peanut seedling growth and partially suppressed southern blight under greenhouse conditions. Positive trends were also observed for several root architecture traits, although these differences were not statistically significant. These findings identify GYB4201^T^ as both a distinct taxonomic entity and a potentially valuable plant-associated bacterial resource. The name *Pseudomonas gilva* sp. nov. is proposed (Table 3).

## Figures and Tables

**Figure 1:**
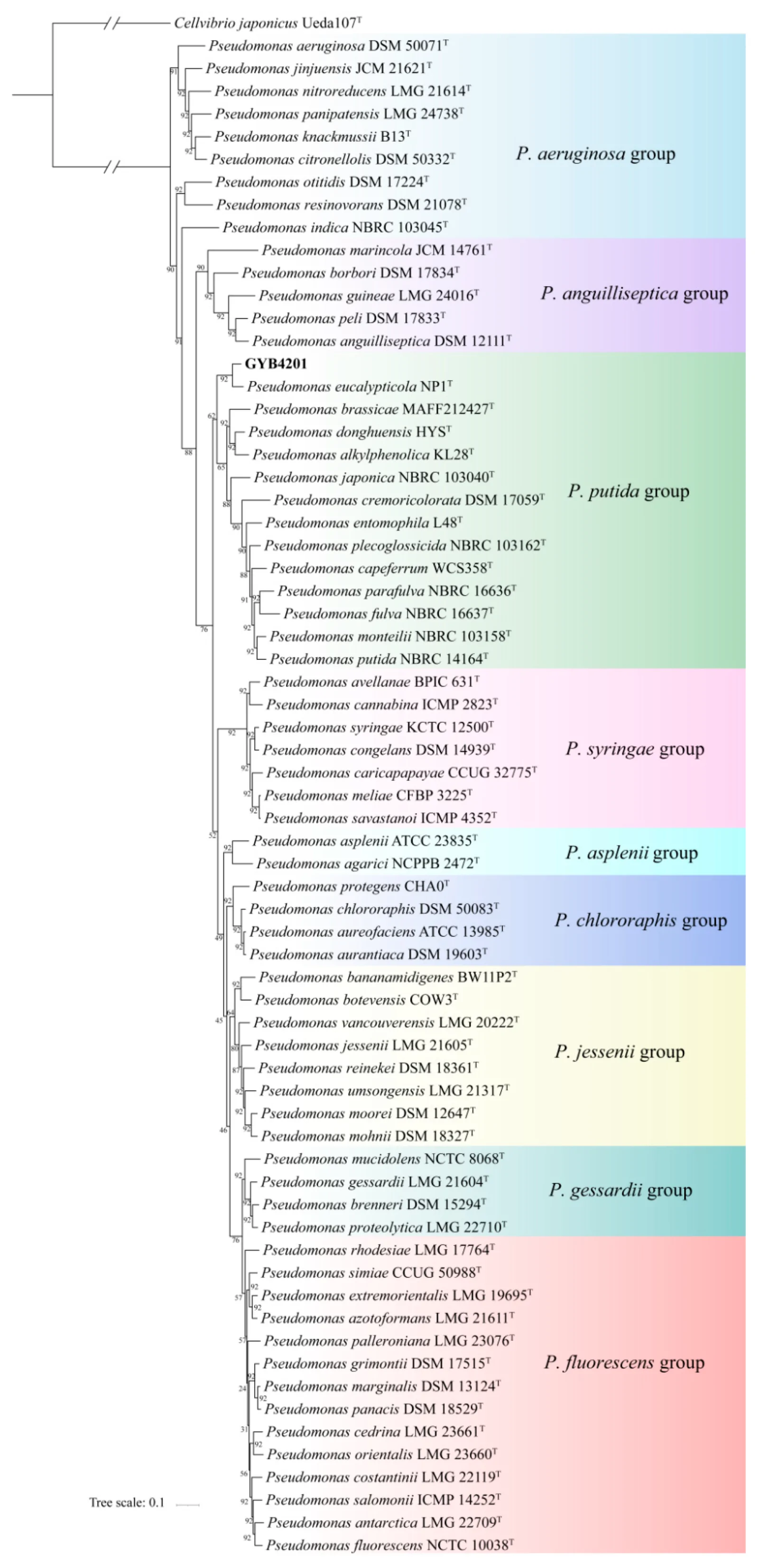
The UBCG phylogenomic tree of GYB4201^T^ and strains in the genus *Pseudomonas* based on 92 conserved bacterial core genes. Strain *Cellvibrio japonicus* was used as an outgroup. The scale bar corresponds to 0.1.

**Figure 2:**
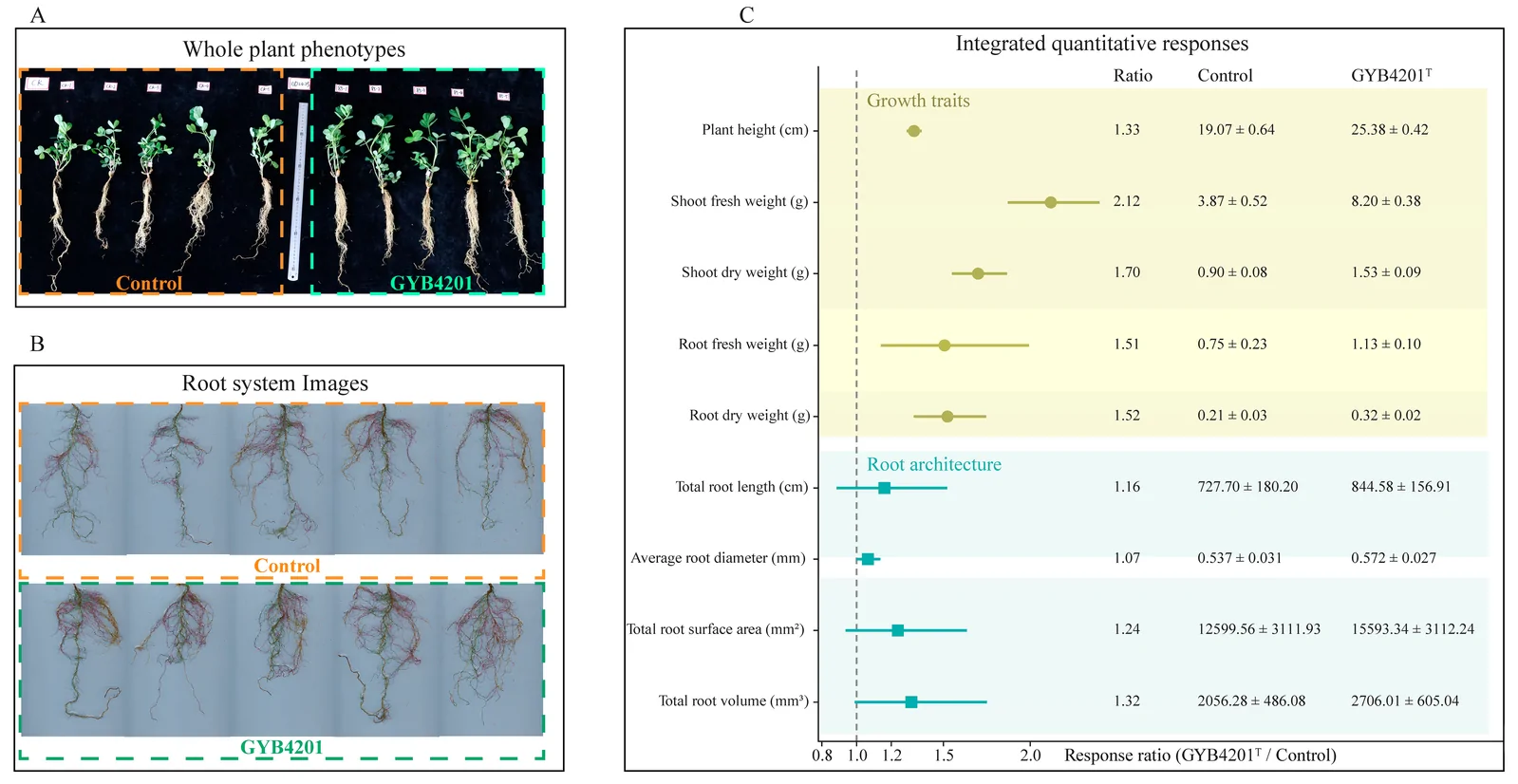
Effects of strain GYB4201^T^ on peanut growth and root system architecture under pot conditions. (**A**) Whole-plant phenotypes of control and GYB4201^T^-treated peanut seedlings at 21 days after inoculation. (**B**) Corresponding root system images obtained by root scanning under identical conditions. (**C**) Integrated quantitative responses of growth and root architecture traits. Points represent treatment-to-control response ratios, horizontal lines indicate 95% confidence intervals, and the vertical dashed line at 1.0 indicates no treatment effect. Circles and light-yellow shading denote growth traits, whereas squares and teal shading denote root architecture traits. Values shown on the right are means ± SD of five biological replicates.

**Figure 3:**
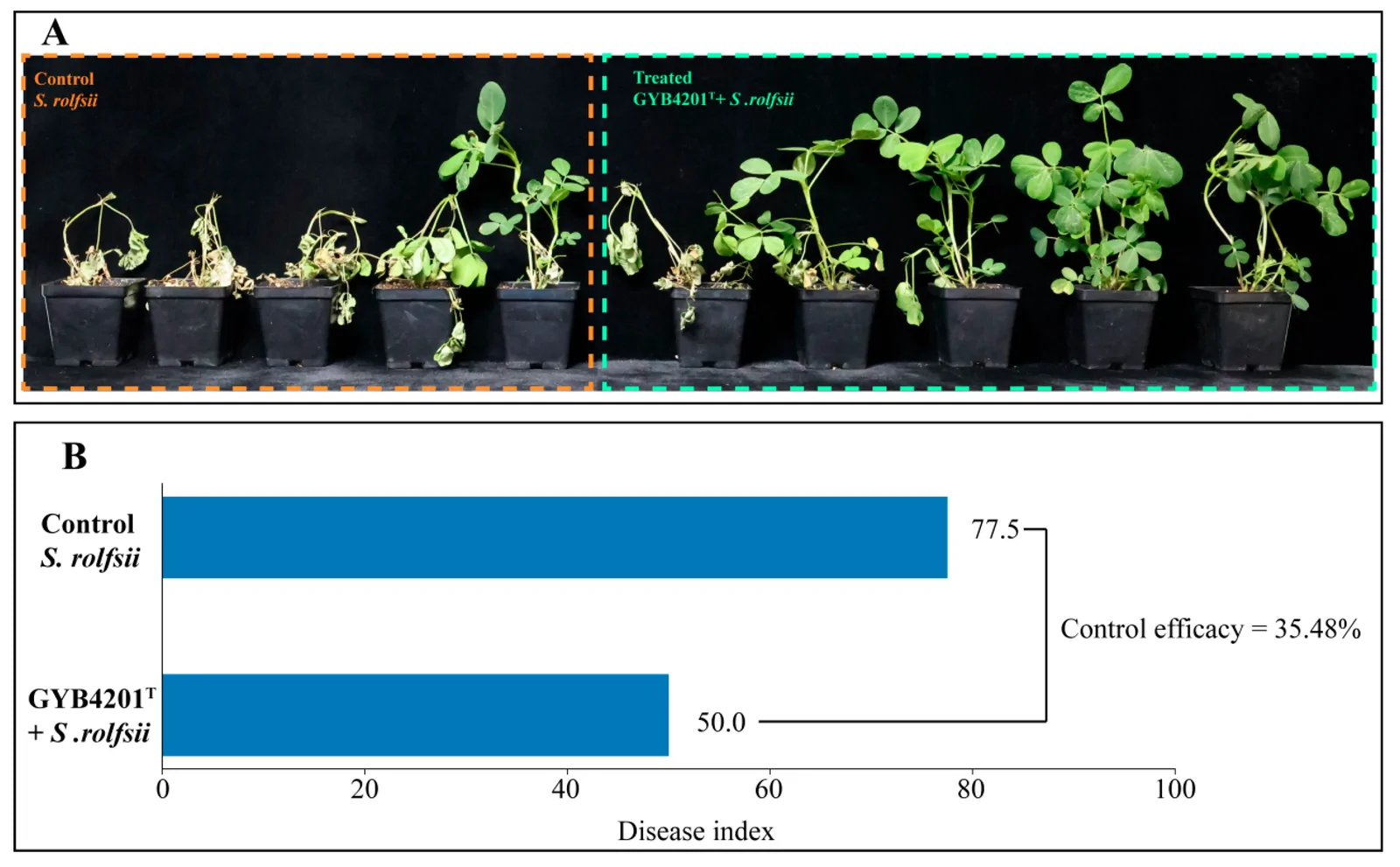
Suppressive effect of strain GYB4201^T^ against peanut southern blight caused by *Sclerotium rolfsii* under greenhouse conditions. (**A**) Representative disease phenotypes of pathogen-inoculated control (CK) and GYB4201^T^-treated peanut plants at 10 days after pathogen inoculation. (**B**) Disease index of the two treatments. The disease index decreased from 77.5 in the pathogen-inoculated control to 50.0 following GYB4201^T^ treatment, corresponding to a control efficacy of 35.48%.

**Table 1:** The similarity of ANI and dDDH between GYB4201^T^ and the closely related strains (%).

	dDDH	GYB4201^T^	RW10^T^	DSM 18327^T^	NP-1^T^	BW11P2^T^	COW3^T^
ANI							
GYB4201^T^			23.50	23.50	32.30	23.10	23.50
RW10^T^		79.40		48.40	23.40	28.40	28.80
DSM 18327^T^		79.43	92.20		23.20	28.30	28.80
NP-1^T^		86.23	79.38	79.15		22.80	22.70
BW11P2^T^		79.62	84.26	84.11	79.00		33.40
COW3^T^		79.88	84.73	84.44	79.16	87.31	

Note: lower left corner represents ANI; upper right corner represents dDDH. RW10^T^, DSM 18327^T^, NP-1^T^, BW11P2^T^, and COW3^T^ represent *P. moorei* RW10^T^, *P. mohnii* DSM 18327^T^, *P. eucalypticola* NP-1^T^, *Pseudomonas bananamidigenes* BW11P2^T^, and *Pseudomonas botevensis* COW3^T^, respectively.

**Table 2:** Differential characteristics of strain GYB4201^T^ and closely related *Pseudomonas* type strains.

Characteristic	1	2	3	4	5	6
Optimum pH	6.5–7	7–7.5	7–7.5	7–8	7	7
Growth in max NaCl (%, *w/v*)	3	4	4	3	5	2.5
Tween 80, L-fucose	W	−	−	−	W	+
N-acetyl-D-glucosamine	−	−	−	−	W	+
D-arabitol, inosine	+	−	−	−	W	+
D-cellobiose, maltose, β-methyl D-glucoside, D-raffinose, D-sorbitol, α-hydroxybutyric acid, Glucose-6-phosphate	+	−	−	−	−	−
D-mannitol, L-aspartic acid, L-glutamic acid, L-serine, γ-aminobutyric acid	+	+	+	−	W	+
D-mannose	+	−	W	+	+	+
D-melibiose	+	−	−	−	W	W
L-rhamnose	W	−	−	+	W	W
D-trehalose	−	−	−	+	−	−
Turanose	−	−	−	W	−	W
Methyl pyruvate	+	+	+	W	−	+
α-keto butyric acid	+	−	−	−	W	−
α-keto glutaric acid	+	+	+	W	W	+
L-alanine	+	W	+	−	W	+
L-histidine	+	+	−	−	+	+
G + C content (mol %)	64.7	59.7	59.6	63.1	60.6	61.2

Strains: 1, GYB4201^T^; 2, P. moorei RW10^T^; 3, P. mohnii DSM 18327^T^; 4, P. eucalypticola NP-1^T^; 5, Pseudomonas bananamidigenes BW11P2^T^; 6, Pseudomonas botevensis COW3^T^. +, Positive; −, negative; W, weakly positive.

**Table 3:** Description of *Pseudomonas gilva* sp. nov.

Parameter	Species
Specific epithet	gilva
Status	sp. nov.
Etymology	gil’va. L. fem. adj. gilva, pale yellow, referring to the colony colour
Description of the new taxon and diagnostic traits	Cells are Gram-stain-negative rods measuring 1.8–2.3 × 0.9–1.2 µm. Growth is strictly aerobic, and catalase and oxidase activities are present. After incubation on TSA at 28 °C for 48 h, colonies are pale yellow, circular and smooth. Growth is observed at 4–35 °C, at pH 4.0–8.0 and in the presence of up to 3% (*w*/*v*) NaCl, with optimal growth at 28–30 °C and pH 6.5–7.0. In Biolog GN2 tests, α-cyclodextrin, dextrin, Tween 40, N-acetyl-D-glucosamine, N-acetyl-D-galactosamine, xylitol, D-fructose, erythritol, gentiobiose, D-galactose, myo-inositol, lactose, α-D-lactose, sucrose, turanose, D-trehalose, acetate, D-glucosaminic acid, p-hydroxyphenylacetic acid, propionate and bromosuccinate are not utilized; Tween 80, L-fucose and L-rhamnose are weakly utilized; the remaining tested substrates are utilized. The cellular fatty acid profile is dominated by C_16:0_, summed feature 8 and summed feature 3. Phosphatidylethanolamine, phosphatidylglycerol and diphosphatidylglycerol constitute the principal polar lipids, and Q-9 is the predominant respiratory quinone.
Country of origin	China
Region of origin	Panzhihua, Sichuan
Date of isolation	05/04/2021
Source of isolation	Rhizosphere soil
Sampling date	05/04/2021
Latitude	26°31′27.12″ N
Longitude	101°46′44.39″ E
16S rRNA gene accession number	PZ169634
Genome accession number	JBWGIG000000000
Genome status	Draft
Genome size	7,050,342 bp
G + C%	64.65
Number of strains in the study	1
Designation of the type strain	GYB4201^T^
Strain Collection Numbers	CFCC 17085^T^ = ICMP 25013^T^

## Data Availability

The GenBank/EMBL/DDBJ accession number for the 16S rRNA gene is PZ169634, and the accession number for the genome sequence of strain GYB4201^T^ is JBWGIG000000000.

## Supplementary Materials

The following supporting information can be downloaded at: https://www.mdpi.com/article/10.3390/microorganisms14102291/s1, Figure S1. Neighbor-joining tree showing phylogenetic relationships among the strain GYB4201^T^ in genus *Pseudomonas* based on 16S rRNA gene sequences. The strain Cellvibrio japonicus was used as outgroup. The scale bar corresponds to 0.01 substitutions per nucleotide site. Filled circles indicate branches recovered by maximum likelihood method and open circles at branches recovered by the maximumparsimony method. Figure S2. The concatenated protein phylogenetic tree among GYB4201^T^ and strains in genus *Pseudomonas* based on a concatenated alignment of 765 ubiquitous single-copy proteins. Figure S3. The polar lipid of GYB4201^T^. Figure S4. Plant-associated functional traits of strain GYB4201^T^. Table S1. High-quality genomes of strain GYB4201ᵀ and selected reference-type strains used for phylogenomic and genome-based relatedness analyses. Table S2. The difference of fatty acid between GYD4201^T^ and the closely related strains.

## Abbreviations

The following abbreviations are used in this manuscript:

ANI	average nucleotide identity
dDDH	digital DNA–DNA hybridization
