# Supplementary material for: Pseudomonas gilva sp. nov., a Plant-Associated Bacterial Species with Growth-Promoting Traits and Partial Suppression of Peanut Southern Blight
Source: Microorganisms. 2026 Oct 8;14(10):2291. doi: 10.3390/microorganisms14102291 (PMC13707960; doi:10.3390/microorganisms14102291)
Supplement: Supplementary file 1 [file microorganisms-14-02291-s001.zip › microorganisms-4556484-supplementary/Supplementary file.pdf]

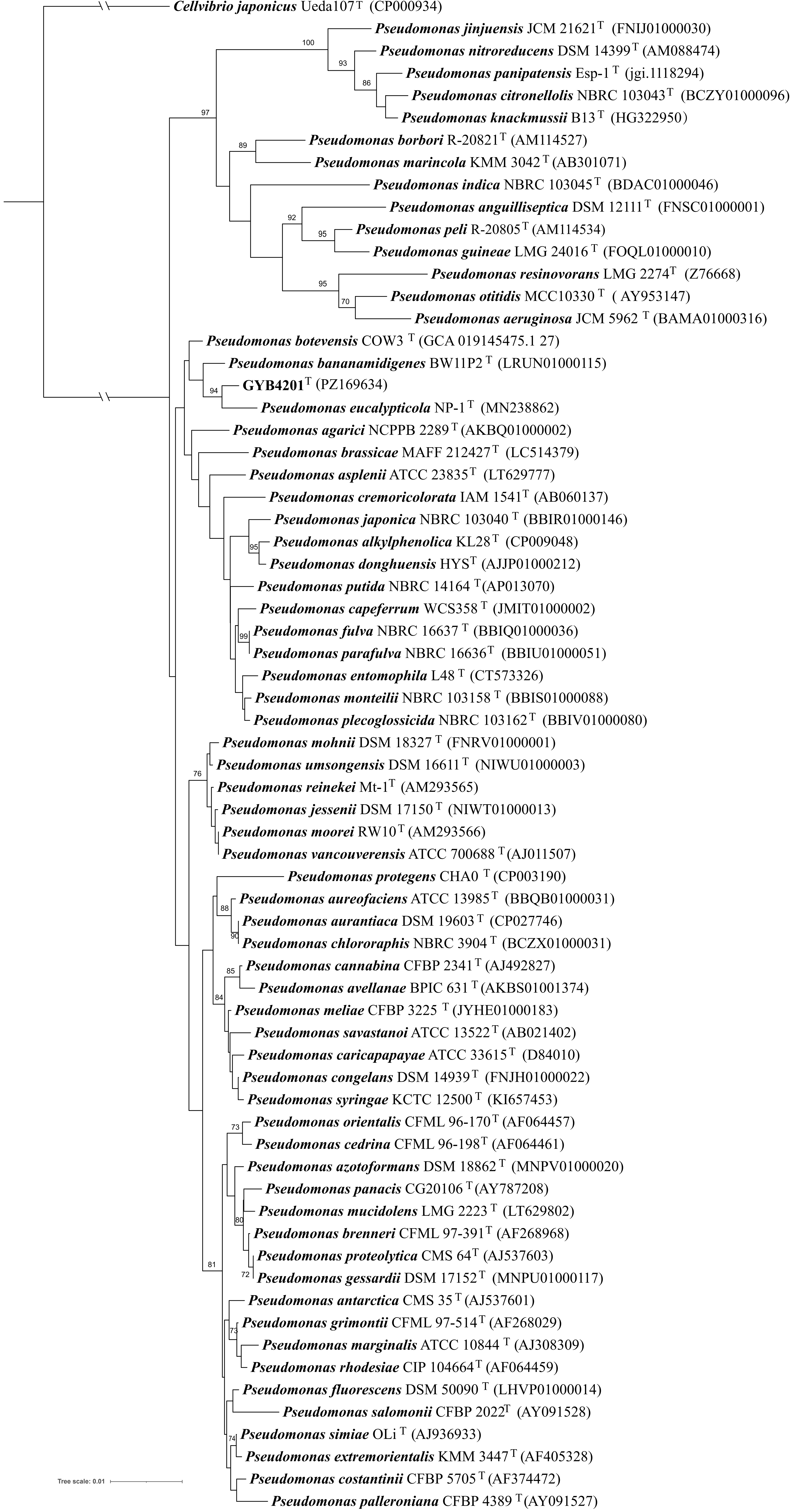

Figure S1. Neighbor-joining tree showing phylogenetic relationships among the strain GYB4201^T in genus *Pseudomonas* based on 16S rRNA gene sequences. The strain Cellvibrio japonicus was used as outgroup. The scale bar corresponds to 0.01 substitutions per nucleotide site. Filled circles indicate branches recovered by maximum likelihood method and open circles at branches recovered by the maximumparsimony method

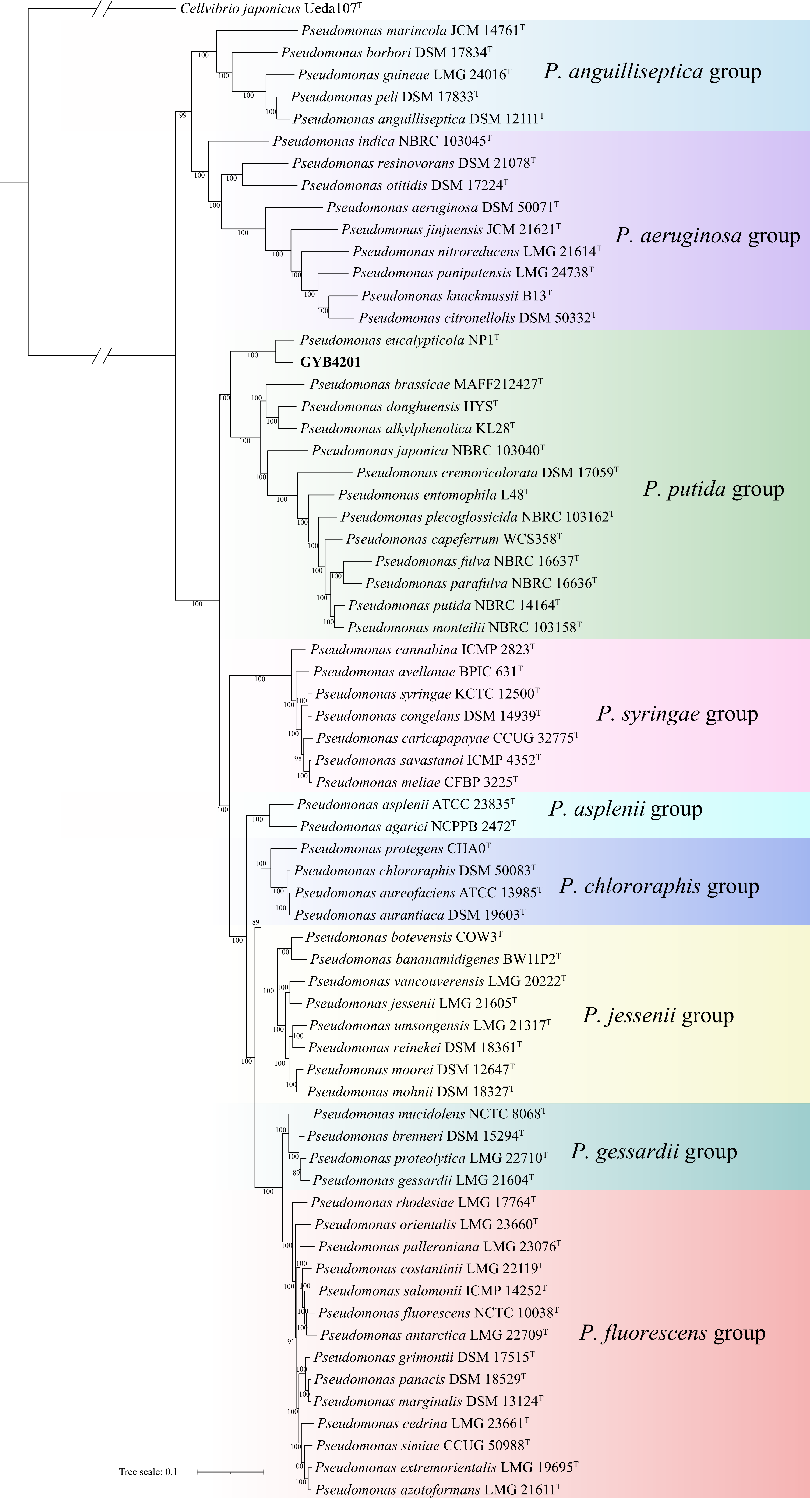

Figure S2. The concatenated protein phylogenetic tree among GYB4201^{T} and strains in genus *Pseudomonas* based on a concatenated alignment of 765 ubiquitous single-copy proteins

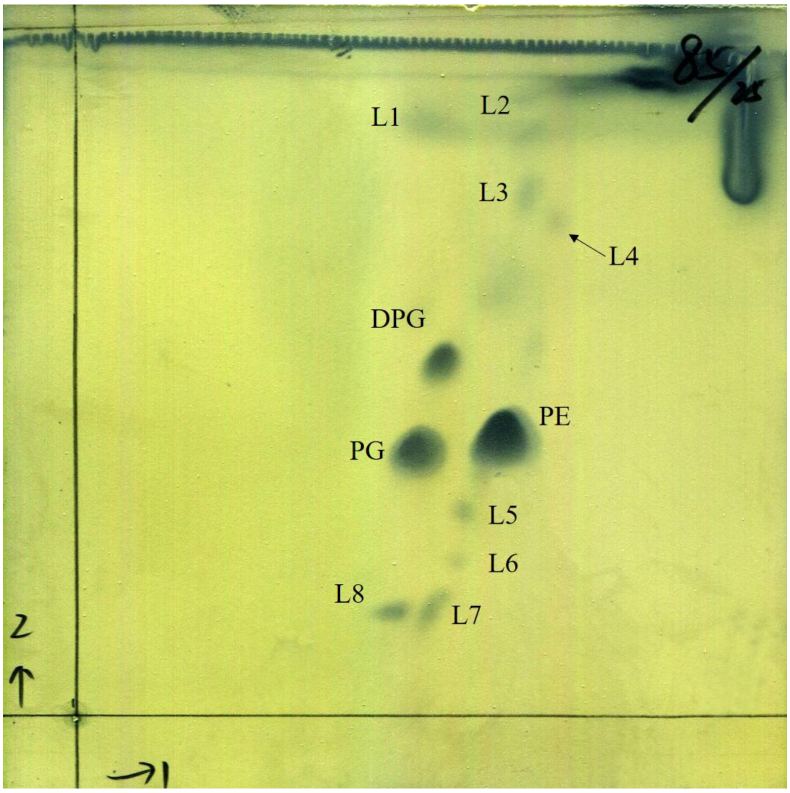

Figure S3. The polar lipid of GYB4201^T.

Note: PE, phosphatidylethanolamine; PG, phosphatidylglycerol; DPG, diphosphatidylglycerol, PL, phospholipid; L, unidentified lipid.

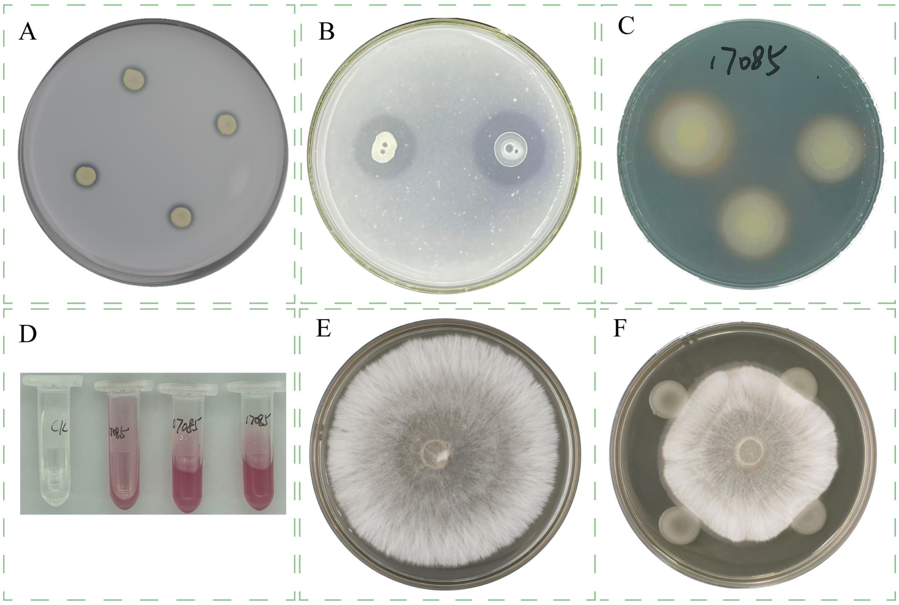

Figure S4. Plant-associated functional traits of strain GYB4201^T.

Note:A, Inorganic phosphate solubilization; B, Potassium solubilization; C, Siderophore production on CAS agar; D, Indole-3-acetic acid (IAA) production; E, normal growth of Sclerotium rolfsii on the control plate; F, inhibition of S. rolfsii by strain GYB4201^T in a dual-culture assay.

Table S2 The difference of fatty acid between GYD4201^{T} and the closely related strains

Fatty acid	GYD4201	RW10^{T}	DSM 18327^{T}	NP-1^{T}
$C_{10:0}$ 3-OH	9.4	2.3	3.9	3.0
$C_{12:0}$	-	3.2	3.5	2.1
$C_{12:0}$ 2-OH	5.1	4.6	3.7	4.6
$C_{12:0}$ 3-OH	6.6	0.8	4.2	4.4
$C_{16:0}$	23.5	35.1	33.2	32.3
$C_{16:1}$*w*7c	-	15.3	23.9	-
$C_{17:0}$ cyclo	4.1	22.8	11.7	19.0
$C_{18:0}$	0.6	-	0.7	0.5
$C_{18:1}$*w*7c	-	10.2	12.5	-
$C_{19:0}$ cyclo *w*8c	0.6	-	-	5.9
Summed Feature 3	17.9	-	-	10.6
Summed Feature 8	20.5	-	-	15.4

Note: Summed Feature 3, $C_{16:1}$*w*7c/$C_{16:1}$*w*6c; Summed Feature 8, $C_{18:1}$*w*7c/$C_{18:1}$*w*6c. RW10^{T}, DSM 18327^{T}, NP-1^{T} represent *P. moorei* RW10^{T}, *P. mohnii* DSM 18327^{T}, *P. eucalypticola* NP-1^{T}, respectively.
